# Three‐dimensional position of impacted maxillary canines: Prevalence, associated pathology and introduction to a new classification system

**DOI:** 10.1002/cre2.151

**Published:** 2018-12-19

**Authors:** Koenraad Grisar, Frederik Piccart, Ali S. Al‐Rimawi, Isabela Basso, Constantinus Politis, Reinhilde Jacobs

**Affiliations:** ^1^ OMFS IMPATH Research Group, Department of Imaging and Pathology, Faculty of Medicine, University Leuven, Department of Oral and Maxillofacial Surgery University Hospitals Leuven Belgium; ^2^ Department of Dental Medicine Karolinska Institutet Sweden

**Keywords:** canine, CBCT, classification, cuspid, impaction, maxillary

## Abstract

Classification of impacted maxillary canines facilitates interdisciplinary communication. Cone beam computed tomography (CBCT) has proven to be superior for the localization of impacted maxillary canines compared with 2D imaging. The purpose of this study was to retrospectively classify a cohort of impacted maxillary canines, using a new developed 3D classification for impacted maxillary canines that is easy to use and does not require complex analysis of the 3D images. A retrospective cohort study was designed, containing CBCT data of 130 patients (male/female: 48/82; median age 16) with a total of 162 impacted maxillary canines. The proposed classification was based on four criteria: vertical crown position, mesiodistal tooth postion, bucco‐lingual crown position, and associated pathology. For all included patients, classification criteria were identified and correlated to treatment selection using a newly developed 3D classification. The most common positions were vertical crown position at apical one third of neighboring teeth, mesiodistal tooth angulation, and palatal crown position. The most frequent associated pathologies were dilaceration of the root and resorption of a neighboring tooth. Significant associations among classification variables and treatment options were observed. Limitations of this study are the retrospective design. CBCT enabled 3D assessment of impacted maxillary canines allowing a classification system that may have an impact on further treatment strategies.

## 1 INTRODUCTION

Impacted maxillary canines are relatively common. When not considering the third molar, the maxillary canine is the most frequently impacted tooth (Bishara, 1992; Cooke & Wang, 2006; Ericson & Kurol, 1986). The prevalence of impacted maxillary canines is reported to be in between 0.9% and 3.3% (Bishara, 1992; Cooke & Wang, 2006; Ericson & Kurol, 1986). The maxillary impacted canine is more often located palatally (85%) than labially (15%) (Bishara, 1992; Cooke & Wang, 2006; Ericson & Kurol, 1986; Ferguson, 1990; Grover & Lorton, 1985; Warford, Grandhi, & Tira, 2003). Root dilaceration is reported to be present in up to 59.5% of the cases (da Silva Santos et al., 2014).

Maxillary canines play a key role in facial aesthetics, development of the dental arch, and occlusion. However, impacted maxillary canines are difficult and time consuming to treat. Moreover, they vary greatly in the inclination and location. Untreated partially erupted or impacted canines may result in several complications such as shortening of the dental arch, formation of follicular cysts, canine tooth ankylosis, recurrent infections, pain, internal resorption, external resorption of the canine and adjacent teeth, or combinations of these factors (Alqerban, Jacobs, Lambrechts, Loozen, & Willems, 2009).

Management of impacted maxillary canines requires an accurate localization. Conducting an assessment by a 3D radiographic examination allows the evaluation of several positional factors that are related to the degree of difficulty of the further treatment, such as the exact position relative to neighboring structures and the orientation over the longitudinal, vertical, and horizontal axis of the impacted tooth (Zuccati, Ghobadlu, Nieri, & Clauser, 2006). Diagnosis of associated pathology such as root resorption of the lateral incisors, root dilaceration, or ankylosis will influence further treatment decisions (Bedoya & Park, 2009).

Impacted teeth are reportedly more difficult to treat in adults. Becker stated that the success rate among patients over 30 years of age was 41%, whereas the success rate for those 20 to 30 years of age was 100% (Becker, Chaushu, & Chaushu, 2010).

So far, few studies have suggested 3D classification systems for impacted maxillary canines based upon their radiological position. The intention is, based on these classifications, to allow a quick determination of the degree of difficulty of an impacted maxillary canine, thus impacting any related treatment strategy (Dalessandri et al., 2013; Dalessandri et al., 2014; Jung, Liang, Benson, Flint, & Cho, 2012).

However, these classifications do not consider possible root anomalies, interactions with surrounding anatomical structures, or associated pathology. Moreover, they require multiple measurements and are time consuming.

Given the lack of studies with an easy to use and straightforward cone beam computed tomography (CBCT)‐based classification for impacted maxillary canines, the aim of the present study is to propose a 3D classification of the position of impacted maxillary canines. A secondary objective is to determine a potential association between the proposed classification and further treatment options.

## 2 MATERIAL AND METHODS

### 2.1 Subjects

The study protocol was approved by the Ethics Committee of our Hospital (s number: s53225).

CBCT imaging of the upper jaw, taken at our department between 2012 and 2016, was screened for the presence of impacted maxillary canines. An impacted tooth is one that fails to erupt into the dental arch within a specific time period. In this study, a tooth was considered impacted when completely or partially intraosseous with more than two thirds of its root developed. Patients were 13–40 years of age at the time of the radiographic acquisition. Patients with syndromatic diseases were excluded. No active orthodontic treatment at the time of acquiring CBCT.

Out of the initial group of 4399 CBCT scans, data from 130 patients (48 male and 82 female; age range 13–41 years) with 162 impacted maxillary canines were obtained. Thirty‐two CBCT scans showed bilateral impaction of the maxillary canines. Information on gender, unilateral/bilateral occurrence, side, location, root dilaceration, root resorption of the adjacent teeth, and the other associated local conditions were gathered. The selected impacted maxillary canines were matched to our classification system.

### 2.2 Radiographic evaluation of canine location

CBCT images were obtained with ProMax 3D (Planmeca, Helsinki, Finland), 3D Accuitomo 170 (J. Morita, Kyoto, Japan), or Newtom VGi evo (Newtom, Verona, Italy) according to the normal clinical protocol for the specific indication and related to the specific machine parameters. Images were evaluated in axial, sagittal, and coronal plane using IMPAX software (Agfa, Mortsel, Belgium). In this software, it is possible to scroll through the *x*, *y*, and *z* planes to best locate and report on the issue of interest.

Next to assessing the location of the canine in three dimensions of the CBCT dataset, the index also scores possible root anomalies, ankylosis, and ectopic position. This combination will lead to a proposal for classification and associated treatment plan as well as a proposal on the prognosis in case an easy located canine has one of the before‐mentioned anomalies. The proposed classification system is easy to use and does not require complex analysis of the 3D imaging. In this way, a clinician should be able to perform the classification procedure directly following the clinical assessment of the patient.

### 2.3 The 3D variations of impaction

**Figure 1:**
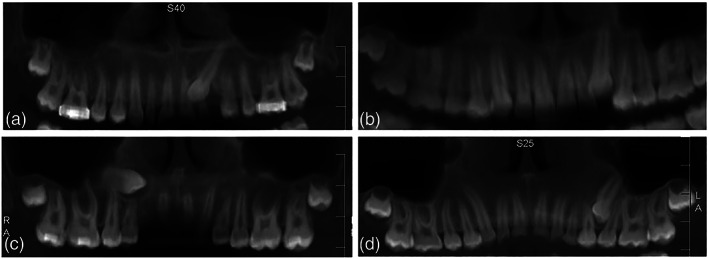
Vertical position of the impacted maxillary canine cusp. (a) Cervical 1/3, (b) middle 1/3, (c) apical 1/3, and (d) supra‐apical

**Figure 2:**
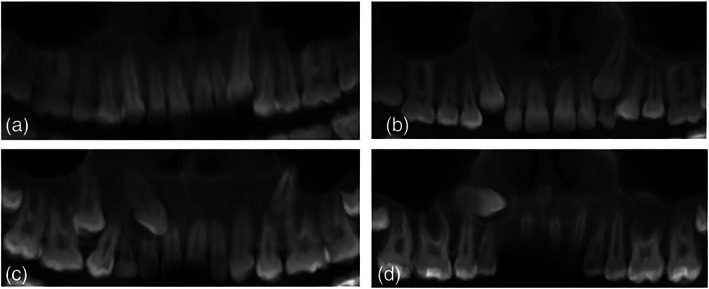
Mesiodistal position of the impacted maxillary canine cusp. (a) Mesiodistal angulation, (b) vertical, (c) horizontal, and (d) transposition

**Figure 3:**
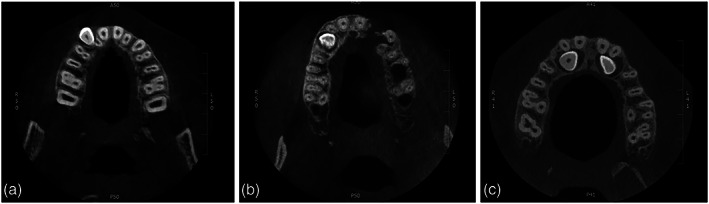
Bucco‐lingual position of the impacted maxillary canine cusp. (a) Vestibular, (b) intra‐alveolar, and (c) palatal

- Vertical position of the canine cusp tip on the *y*‐axis compared with the adjacent teeth. This will be analyzed at the 3D PANORAMIC view (Figure 1).
  - Cusp tip lies in a horizontal plane occlusal to the cemento‐enamel junction of the incisor.
  - Cusp tip lies in a horizontal plane with the cervical third of the incisor root.
  - Cusp tip lies in a horizontal plane with the middle third of the incisor root.
  - Cusp tip lies in a horizontal plane with the apical third of the incisor root.
  - Cusp tip is supra‐apical to the incisor root.
- Mesiodistal position of the canine on the *x*‐axis compared with the adjacent teeth. This will be analyzed at the 3D PANORAMIC view (Figure 2).
  - MD angulation (mesial position crown and distal position apex)
  - DM angulation (distal position crown and mesial position apex)
  - Vertical position
  - Horizontal position
  - Ectopic or inverted position
- Buccopalatal cusp tip position on the *z*‐axis compared with the adjacent teeth. This will be analyzed at the axial views (Figure 3).
  - Vestibular position, outside of the outline as suggested by the neighboring teeth
  - Intra‐alveolar position, within the area as suggested by the vestibular and palatal outlines of the neighboring teeth
  - Palatal position, outside of the outline as suggested by the neighboring teeth

### 2.4 Associated pathology

**Figure 4:**
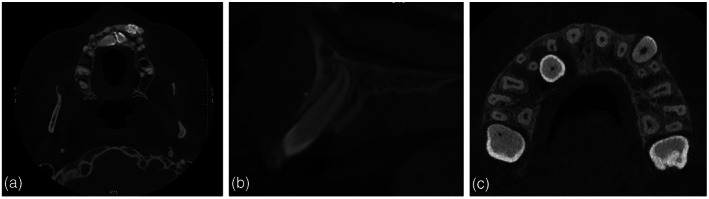
Associated anomalies of the impacted maxillary canine cusp. (a) Odontoma, (b) dilaceration, and (c) resorption of lateral incisor

This will be analyzed at the axial, sagittal, and coronal views (Figure 4).

- Root dilacerations—interaction with surrounding anatomical structures was evaluated.
- Ankylosis
- Relation to neighboring anatomical structures
- Resorption of neighboring teeth
- Presence of odontoma or other local pathology

### 2.5 Applied treatment

The applied treatment for the impacted maxillary canine was recorded by screening clinical records and graded as follows:

- monitoring: with or without removing the primary canine;
- surgical exposure;
- surgical removal; and
- autotransplantation.

### 2.6 Statistical analysis

Data were summarized by means of frequency tables. Relations between the position or treatment on the one hand and (for position) treatment or associated pathology on the other hand were assessed by means of cross‐tabulations and a Fisher exact test.

## 3 RESULTS

### 3.1 Patients and maxillary canine characteristics

Among the 4399 CBCTs screened, impacted maxillary canines were identified in 130 patients with a total of 162 impacted canines. Patient's characteristics are described in Table 1. Patient age ranged from 13 to 41 years (mean age: 18; *SD* +/−6.47). Regarding gender, 48 patients were male (36.9%) and 82 were female (63.1%). In 32 patients, there was bilateral impaction of the maxillary canines. Unilateral impacted maxillary canines were situated almost equally on both right side (*n* = 79; 49%) and left side (*n* = 83; 51%). Distribution of the 162 impacted maxillary canines according to the proposed classification is presented in Table 2. Impacted maxillary canines were most frequently found to be vertically positioned at the middle third of the incisor root (*n* = 79, 48.8%), to have a mesiodistal angulation (*n* = 111; 68.5%) and an intra‐alveolar bucco‐lingual position (*n* = 88, 54.3%). Most frequent associated anomalies were dilaceration of the root (*n* = 29, 17.9%) and resorption of neighboring teeth (*n* = 24, 14.8%). In case of resorption, this was mainly concerning the lateral incisor; 6.8% of the impacted maxillary canines was found to be ankylosed (*n* = 11).

**Table 1:** Characteristics of the patients

Variables	Frequency (*n*)	(%)
Patient age (years)		
13–19	107	82.3
20–29	12	9.2
30+	12	9.2
Sex		
Male	51	39.2
Female	79	60.8
Location		
Unilateral	98	75.4
Bilateral	32	24.6
Right	79	48.8
Left	83	51.2

**Table 2:** Distribution of impacted maxillary canines along 3D classification

Variables	Frequency (*n*)	(%)
Vertical position		
Above the cemento‐enamel junction of the incisor	4	0.6
At the cervical third of the incisor root	17	10.5
At the middle third of the incisor root	79	48.8
At the apicale third of the incisor root	55	34
Supra‐apical	7	4.3
Mesiodistal position		
Mesiodistal angulation	111	68.5
Disto‐mesial angulation	0	0
Vertical	28	17.3
Horizontal	22	13.6
Ectopic or inverted	1	0.6
Bucco‐lingual position		
Vestibular	24	14.8
Intra‐alveolar	88	54.3
Palatal	50	30.9
Associated anomalies		
Ankylosis	11	6.8
Dilaceration	29	17.9
Association with the nasal cavity	10	34.5
Association with the floor of the sinus	8	27.6
Association with the roots of the first premolar	2	6.9
No association	9	31
Resorption	24	14.8
Central incisor	2	8.3
Lateral incisor	19	79.2
Premolar 1	4	17
Premolar 2	1	4.2
Odontoma	3	1.9

### 3.2 Association between teeth position and anomalies

We observed significant relations between teeth position and anomalies, considering vertical position and ankylosis, and between mesiodistal and bucco‐lingual position and dilaceration. Ankylosis was more frequently observed in case of high vertical position above CEJ (two cases, *p* < 0.05). Dilaceration was more often observed in case of horizontal position or mesial angulation (4 and 27 cases, *p* = 0.05). Dilaceration was mostly observed in case of palatal position (22 cases, *p* < 0.05).

### 3.3 Treatment

In 46 patients (58 impacted maxillary canines), the further treatment plan was not reported in the medical records, considering referral by external orthodontist for imaging only. In the group with complete patients records (84 patients and 104 impacted maxillary canines), following treatment modalities were reported: surgical exposure of the impacted maxillary canine (*n* = 59, 56.7%), autotransplantation (*n* = 19, 18.3%), removal (*n* = 15, 14.2%), and watchful waiting with or without removal of the primary canine (*n* = 11, 14.2%). Treatment options are summarized in Table 3.

**Table 3:** Treatment choices

Variables	Frequency (*n*)	(%)
Monitoring with or without removing the primary canine	11	10.6
Surgical exposure	59	56.7
Surgical removal	15	14.2
Autotransplantation	19	18.3

### 3.4 Association between teeth position and treatments

The associations between choice of treatment and each of the classification variables were also evaluated (Table 4). We only observed a significant relation between mesiodistal position and treatment option: In case of horizontal position of the impacted maxillary, canine autotransplantation was most often preferred as the treatment choice (10 cases, *p* < 0.05). For impacted canines with mesioangulation or vertical position, surgical exposure and traction was the treatment of choice (43 and 10 cases, *p* < 0.05). There was only one case of transposition of the impacted maxillary canine, and there, the clinician opted for a surgical removal of the canine involved.

**Table 4:** Association between treatment choices and classification variables

	Spontaneous eruption	Transplantation	Removal	Surgical exposure	Spontaneous eruption (%)	Transplantation (%)	Removal (%)	Surgical exposure (%)
Mesiodistal position								
Horizontal (*n*)	0*	10*	3*	6*	0*	52.6*	15.8*	31.6*
Mesioangulation (*n*)	10*	7*	11*	43*	14.1*	9.9*	15.5*	60.6*
Transposition (*n*)	0	0	1	0	0	0	100	0
Vertical (*n*)	1*	2*	0*	10*	7.7*	15.4*	0*	76.9*
Horizontal (%)	0	52.6	20	10.2				
Mesioangulation (%)	90.9	36.8	73.3	72.9				
Transposition (%)	0	0	6.7	0				
Vertical (%)	9.1	10.5	0	16.9				
Ankylosis								
Yes (*n*)	0	3	1	1	0	60	20	20
No (*n*)	11	16	14	58	11.1	16.2	14.1	58.6
Yes (%)	0	15.8	6.7	1.7				
No (%)	100	84.2	93.3	98.3				

*Note*. Significant results are marked with:

*

No significant association could be oberved between choice of treatment and vertical of bucco‐lingual position or associated anomalies.

## 4 DISCUSSION

Most of the literature on classification of impacted maxillary canines contains results based on 2D images. Recently suggested 3D classifications do not consider possible root anomalies, interactions with surrounding anatomical structures, or associated pathology. Moreover, they require multiple measurements and are time consuming.

The aim of this study was to propose an alternative 3D classification system of the position and possible associated anomalies of impacted maxillary canines.

A preoperative CBCT examination is considered an important assessment tool for planning the treatment of impacted maxillary canines and for choosing the treatment. Some important findings that may affect this choice can only be obtained from CBCT images and not from 2D images. Among them is the bucco‐lingual position, the real proximity of the roots to the floor of the sinus or nasal cavity, anatomy of the apical part of the root, signs of ankylosis, or root resorption of neighboring teeth (Alqerban et al., 2009; Alqerban, Jacobs, Fieuws, & Willems, 2011; Alqerban, Storms, Voet, Fieuws, & Willems, 2016).

In our population characteristics, we found that most of our patients were 19 years or younger (82.3%). This is to be expected when investigating impacted maxillary canines because most of the patients will receive orthodontic or surgical treatment in this age group.

When we consider gender, we observe that there is a striking higher frequency female patients within our population. This is in line with the findings in the current literature (da Silva Santos et al., 2014).

Most of the cases were unilateral, and there was an equal left right distribution. Regarding the distribution of the impacted maxillary canines along our newly suggested classification, we observed that most of the teeth were found intra‐alveolar positioned in a mesiodistal angulation with the cusp in the same horizontal plane as the middle third of the incisor root.

Prevalence of ankylosis (14.8%), dilacerations (17.9%), resorption of neighboring teeth (14.8%), or odontoma (1.9%) were comparable with other reports in the current literature (Becker, Smith, & Behar, 1981; Botticelli, Verna, Cattaneo, Heidmann, & Melsen, 2011; da Silva Santos et al., 2014; Dachi & Howell, 1961; Ericson & Bjerklin, 2001; Lai, Suter, Katsaros, & Bornstein, 2014; Oliver, Mannion, & Robinson, 1989; Walker, Enciso, & Mah, 2005).

When considering the relation between the position of the impacted maxillary canine and the choice of treatment, we observed a significant difference evaluating mesiodistal position. Horizontal position was more frequently associated with autotransplantation of the maxillary canine. In case of mesioangulation or vertical position, surgical exposure and traction were the treatment of choice. This is as expected, considering that autotransplantation is mostly associated with a more complex localization of the impacted maxillary canine.

Future studies should investigate the relationship of this classification system and treatment outcomes as such that a scoring system can be associated for prediction of treatment duration, risks, and success rate. This would be helpful in the management for patients with impacted maxillary canines. It would also help in correctly estimating the costs of the treatment involved.

## 5 CONCLUSIONS

Planning of impacted maxillary canine treatment should be based on 3D images. With CBCT, it is possible to correctly define the position of the impacted maxillary canine and to recognize accompanying abnormalities such as ankylosis, dilaceration of the root with or without anchorage to the floor of the sinus or nasal cavity, resorption of neighboring teeth, or odontoma.

The present study proposes the use of a standardized classification system, aiding identification of more challenging cases. The proposed classification system is easy to use clinically, allowing assessment and decision for further treatment following patient examination. In the long run, this classification may eventually be able to predict outcome expectations.

## CONFLICT OF INTEREST

The authors report no conflicts of interest related to this study.

## Footnotes

*
*p* < 0.001.
